# Removal of migrated pancreatic plastic stent by screw-type device under enteroscopy in pancreaticoduodenectomy

**DOI:** 10.1055/a-2723-7656

**Published:** 2025-11-04

**Authors:** Mitsuru Okuno, Fumiya Kataoka, Tsuyoshi Mukai, Hiroshi Araki, Eiichi Tomita, Hisataka Moriwaki, Masahito Shimizu

**Affiliations:** 1 73505 Department of Gastroenterology, Matsunami General Hospital, Gifu, Japan; 2 First Department of Internal Medicine, Gifu University Hospital, Gifu, Japan

Pancreatic drainage using a plastic stent is effective for treating pancreaticointestinal anastomotic strictures after pancreaticoduodenectomy. We report a case of troubleshooting for removal of a stent that migrated to the distal end of the main pancreatic duct (MPD) using a screw-type dilator (Tornus ES; Olympus Medical Systems, Tokyo, Japan).

A 71-year-old woman with a history of pancreaticoduodenectomy for distal cholangiocarcinoma 1 year earlier presented with abdominal pain. Computed tomography revealed a dilated MPD and pancreatitis ( Fig. 1 **a** ). Endoscopic retrograde pancreatography using an enteroscope was performed for a suspected pancreaticointestinal anastomotic stricture. A 5-Fr pancreatic plastic stent (PS) was placed at the anastomosis site, relieving pancreatitis ( Fig. 1 **b** ). Four months later, scheduled enteroscopic PS exchange was performed. The PS was replaced with one of the same size; however, severe intestinal deformation and insufficient anastamotic distance caused accidental migration into the MPD. Although grasping forceps, biopsy forceps, and basket catheters were inserted into the MPD to remove the migrated PS, none of the attempts resulted in PS recovery or rather pushed the PS further into the distal end of the MPD. As the guidewire remained inside the PS, a screw-type dilator (Tornus ES) was advanced into the PS under its guidance. After rotating the Tornus to screw it into the PS, the migrated PS was successfully removed along with the Tornus ES ( Fig. 2 , Video 1 ).

**Fig. 1:**
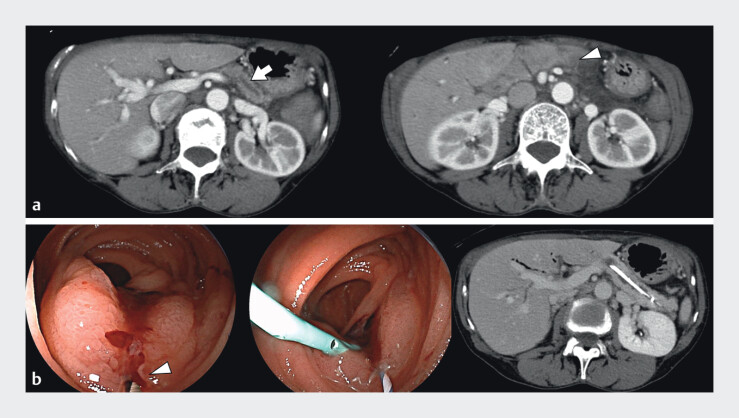
**a** Computed tomography revealed a stricture of the pancreaticointestinal anastomosis (arrowhead) and pancreatitis. The main pancreatic duct was dilated (arrow). **b** Enteroscopy revealed a stricture of the pancreaticointestinal anastomosis (arrowhead). A pancreatic plastic stent was placed at the anastomosis, successfully treating the pancreatitis.

**Fig. 2:**
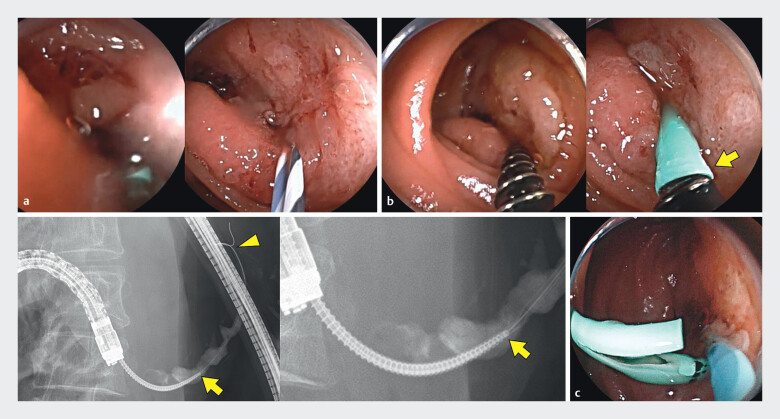
**a** Straight-type 5-Fr pancreatic PS was placed for pancreaticointestinal anastomosis. However, the plastic stent accidentally migrated into the main pancreatic duct of the patient. **b** As the guidewire remained in the PS, a screw-type dilator (Tornus ES; Olympus Medical Systems, Tokyo, Japan) was advanced into the PS under guidewire guidance. After rotating the Tornus to screw it into the PS, the migrated PS (arrowhead) was successfully removed along with the Tornus ES (arrow). **c** The migrated PS was replaced at the pancreaticointestinal anastomosis site. PS, plastic stent.

Successful removal of a migrated 5-Fr pancreatic plastic stent using a screw-type dilator.Video 1

Although various devices, including forceps and basket catheters, have been reported to remove the migrated pancreatic PS 1 2 , in this case, they failed due to the V-shaped MPD and PS location, compounded by limited enteroscope maneuverability after reconstruction. The screw-type dilator, easily inserted into the MPD and then into the PS over the guidewire, allowed capture from the inside by screwing. When the guidewire is within the PS, a screw-type dilator may be an effective option for retrieval.

Endoscopy_UCTN_Code_CPL_1AK_2AD

## Notes

Endoscopy E-Videos https://eref.thieme.de/e-videos
*E-Videos* is an open access online section of the journal *Endoscopy* , reporting on interesting cases and new techniques in gastroenterological endoscopy. All papers include a high-quality video and are published with a Creative Commons CC-BY license. Endoscopy E-Videos qualify for HINARI discounts and waivers and eligibility is automatically checked during the submission process. We grant 100% waivers to articles whose corresponding authors are based in Group A countries and 50% waivers to those who are based in Group B countries as classified by Research4Life (see: https://www.research4life.org/access/eligibility/ ). This section has its own submission website at https://mc.manuscriptcentral.com/e-videos .
